# Supplementary material for: Chromothripsis during telomere crisis is independent of NHEJ, and consistent with a replicative origin
Source: Genome Res. 2019 May;29(5):737–49. doi: 10.1101/gr.240705.118 (PMC6499312; doi:10.1101/gr.240705.118)
Supplement: Supplemental Material [file supp_gr.240705.118_Supplemental_file_1.zip › contigs/annotated_contigs/DB53/contig.2.DB53_length_730_mean_cov_13.2164383562.docx]

**DB53_length_730_mean_cov_13.2164383562**

```
GGGATCTTCACGCTCATGACCTCATCTAAACCAAATTATCTACCAAAGGCCCCACCTTCAAATATCATAACATTAGGGGTTAGGGCTTC
 >chr8:85432974-85433396 - E=4e-242
AATATATGAATTTTGAGGAGACATATTTCAATCCACAGCAGTTCATATTATCCAACAGTAAGAACCCACTTTTCGTTTTGAAGCTTTGG

GGAAATGGAATAAGCTCCCTCTGAGCCATAAGAGGCAATGTGATTGTTGCTATATTTATTCTGTGAGGGCTCGTGTAAACCAGTGGTCC

CCAAACTGTTTGGCACCAGGGACTGATTTCATGGAAGACAATTTTTCCATAGGATGCAGGTGGTGGGACAAGGGTGAGAGATGGTGAGG

TGGTTTCAGGATGAAACCATTCCACCTCAGATCATCAAGCATTAGTTAGATTCTCGTAAGGAGCAC|AATTTGATAATAAAATGTAAGA
                                                                    >chr8:85457824-85458
TATCTTTACTTGTAGATATGACTATATAGTTTGAGAAGCAGAAAATATCAACAGGACCACTTAAGAAAGAGACAACTTGTAAAGTATGT
132 - E=2e-170
CATTATAATATTACTCCCCCAAAGCTCAGTAGCATTCATATATGAAAACAAAATCAGTTGTAAGATATAAAGAAAAAAAGATATAATGT

GAGAAAATACTCTATTTATGCTAACAAATTTAAAAGAACGTATTAAAACAGAAACATAATAAATATACATGAGGATCATACATGAAGTA

AACCATAAAACACTTCCAA
```
